# Comparative Analysis of Antioxidant Properties of Honey from Poland, Italy, and Spain Based on the Declarations of Producers and Their Results of Melissopalinological Analysis

**DOI:** 10.3390/nu14132694

**Published:** 2022-06-28

**Authors:** Anna Puścion-Jakubik, Joanna Bielecka, Monika Grabia, Renata Markiewicz-Żukowska, Jolanta Soroczyńska, Dariusz Teper, Katarzyna Socha

**Affiliations:** 1 Department of Bromatology, Faculty of Pharmacy with the Division of Laboratory Medicine, Medical University of Białystok, Mickiewicza 2D Street, 15-222 Białystok, Poland; joanna.bielecka@umb.edu.pl (J.B.); monika.grabia@umb.edu.pl (M.G.); renmar@poczta.onet.pl (R.M.-Ż.); jolanta.soroczynska@umb.edu.pl (J.S.); katarzyna.socha@umb.edu.pl (K.S.); 2 HoneyLab Teper & Waś s. c., Generała Fieldorfa Nila Street, 24-100 Pulawy, Poland; dariusz.teper@honeylab.pl

**Keywords:** DPPH, FRAP, total phenolic content, Pfund scale, color intensity

## Abstract

Natural bee honeys are commonly used by patients for nutritional, preventive, and curative purposes. Honey varieties produced in other countries, including Italy and Spain, are gaining popularity. The aim of the study was to evaluate selected antioxidant properties of honey, taking into account the declared and actual variety. The research material consisted of 105 honey samples, including honeys from Poland (*n* = 50), from Spain (*n* = 35), and from Italy (*n* = 20). The variety was determined by the melissopalinological method, and in the case of honeydew honeys, the electrical conductivity was measured. Total phenolic content (TPC), color intensity, color in Pfund scale, DPPH, and FRAP were assessed. Polish buckwheat honeys, with confirmed botanical origin, are characterized by the highest median of the TPC (213.05 mg GAE/100 g), the highest color intensity (1.138 mAU), and the highest value in the FRAP test (0.394 µM Fe^2+^/mL). In conclusion, proper labeling of bee honeys is necessary so as not to mislead consumers, and buckwheat honeys from Poland can be recommended to patients for prophylactic purposes in order to provide antioxidants in the diet.

## 1. Introduction

Statistical data show that in 2020 there were 2,967,000 hives in Spain, 1,766,000 in Poland, and 1,687,000 in Italy, which makes these three countries among the five European countries with the highest number of bee colonies. China is in first place in terms of honey production, and the production of honey in this country covers as much as 24% of world production. The European Union countries are second in the world (12%) [1].

Melissopalinology is a field of palynology whose aim is the quantitative and qualitative assessment of bee products in terms of pollen grains present in the microscopic specimen [2]. In the case of unifloral honeys, based on percentage pollen grains that are most dominant over other grains or reach the required level characteristic for the variety in the honey sediment, the variety of honey is named after the plant [3,4].

Consumers and patients, selecting varieties of honey, sometimes expect specific properties resulting from the variety. Food available for sale, in accordance with legal requirements, should be properly labeled. Among bee honeys, there is often the problem of incorrect determination of the type of honey by beekeepers, e.g., only on the basis of the color, texture, taste, smell, and observation of flowering and nectar secretion of plants. Data in the literature indicate numerous labeling irregularities. For example, in 2018, the Office of Competition and Consumer Protection, operating in Poland, published the results regarding the evaluation of the quality of honeys. A total of 269 batches of honey were inspected. There were irregularities in the labeling of honeys in the case of linden honeys (the content of the main pollen from *Tilia* spp. was from 1.9 to 7.7%), buckwheat honey (the content of the main pollen from *Fagopyrum* Mill. in the range from 23.8 to 35.5%), dandelion honey (pollen content from *Taraxacum officinale* F.H. Wigg. was found to be 5.1%), and in acacia honey (the share of dominant pollen from *Robinia pseudoacacia* L. was found at the level of 22.2%) [5].

Due to the large number of samples of incorrectly labeled honeys and the time-consuming nature of the classic method, alternative methods are sought. An example is the automatic pollen analysis based on the image analysis technique. This method is based on the conversion of visual information into mathematical descriptions. For this purpose, inter alia, 2D and 3D morphological features, color, and texture are considered [6].

Data in the literature indicate promising antioxidant properties of honey, which can be used in the prophylaxis of many diseases. For example, Manuka honey from New Zealand has antioxidant, antiproliferative, and antibacterial properties, and can inhibit the process of carcinogenesis by influencing various molecular processes [7]. With the use of *Glioblastoma multiforme* U87MG cell cultures, it was shown that honeys from Poland are a promising factor with anti-proliferative and anti-metastatic properties [8]. Polyphenols present in honey contribute to the occurrence of overlapping mechanisms of chemopreventive action in multi-step carcinogenesis, including by inhibiting mutagenesis or inducing apoptosis. The antibacterial effect of honey is explained by, among other factors, content of flavonoids [9], defensin-1, and methylglyoxal. Another benefit involves a protective effect on the cardiovascular system [10].

Due to the growing interest of people in consuming honey for prophylactic and therapeutic purposes, as well as openness to products from other countries, the aim of the research was to evaluate selected antioxidant properties of honeys from Poland, Italy, and Spain, taking into account the variety declaration (provided by beekeepers) and the proper variety (the result of melissopalinological analyses).

## 2. Materials and Methods

### 2.1. Materials

The study included 105 samples of natural bee honeys available for sale in onsite and online stores in Poland. The research covered the most common bee honey varieties available for sale, from five different producers. Fifty honeys were obtained from apiaries in Poland, 35 in Spain, and 20 in Italy—five honeys of each variety. The names of the varieties were specified by beekeepers on the labels. Characteristics of the varieties are presented in Table 1.

### 2.2. Methods

#### 2.2.1. Determination of Variety

The basis of the method was developed by Louveaux et al. [11]. In order to determine the honey variety, 10 g of honey (accuracy 0.001 g) were weighed into a Falcon conical tube, and water at 50 °C was added. The resulting solution was centrifuged for 10 min (3000 rpm), then the supernatant was removed and the analytical steps were repeated. The solution was then withdrawn with a pipette, leaving a suitable layer of liquid above the sediment, and mixed until a suspension was obtained, from which a microscope preparation was prepared. Then, using an optical microscope at 400 times magnification, at least 300 consecutive pollens were classified into botanical species, excluding pollen from nectarless and anemophilous plants. The obtained results were converted into percentages. At least two repetitions were made for each honey sample [12].

#### 2.2.2. Determination of Water Content

The water content in bee honey corresponds to the refractive index, which is determined by the refractometric method. For this purpose, 5 g were weighed and melted in a water bath (Grant, A-BioTech, Wroclaw, Poland) at 40 °C. Then the honey was placed on the prism of the refractometer and the refractive index was read, and corrected for the ambient temperature. The water content is shown as % [12]. The determination of the water content was used to calculate the mass of honey that was dissolved to determine the electrical conductivity.

#### 2.2.3. Determination of Electrical Conductivity

Then, based on the water content, the quantities of honey were calculated. Solutions were prepared which were brought to 20 °C and their electrical conductivity was measured. The result was then multiplied by the constant value, characteristic for the conductivity electrode. The results are expressed in mS/cm [12].

#### 2.2.4. Determination of Total Phenolic Content

The determination of the total phenolic content (TPC) was carried out on the basis of the methodology developed by Zhou et al. [13], including our own modifications. Honey was weighed out (1 g), to which 9 mL of distilled water have been added. The dissolved sample was centrifuged for 5 min at 2000 rpm. Next, 0.25 mL of supernatant was taken, 1.25 mL of 0.2 N Folin–Ciocalteu reagent was added thereto, mixed for 5 min and 1 mL of Na_2_CO_3_ solution was added. Samples were kept in the dark for 120 min and the absorbance at 760 nm was measured against water, using the U-2001 spectrometer (Hitachi, Tokyo, Japan). Gallic acid solutions (Sigma-Aldrich, St. Louis, MO, USA) were used to obtain the calibration curve. The results were expressed as gallic acid equivalents (GAE) per 100 g.

#### 2.2.5. Determination of Color Intensity (CI)

The principle of this method is based on the measurement of the color intensity, which comes from antioxidant components, including carotenoids and flavonoids [14].

In order to determine the color intensity, 5 g of honey was weighed (with an accuracy of 0.001 g), and water at 45 °C was added to the volume of 10 mL. The solutions were then sonicated in ultrasonic washer for 5 min and filtered through 0.45 µm syringe filters. In the next step, the absorbance of the solutions was measured against water at 450 and 720 nm using U-2001 spectrometer (Hitachi, Tokyo, Japan). Three determinations were made for each honey. The results are expressed in mAU [14].

#### 2.2.6. Determination of Color in Pfund Scale (CP)

The determination consisted in weighing out 5 g of the sample (with an accuracy of 0.001 g) and dissolved up to 10 mL in distilled water. The solutions were placed in a water bath at 50 °C to dissolve the sugar crystals, and then the absorbance, against water, was measured at 635 nm using U-2001 spectrometer (Hitachi, Tokyo, Japan). The numerical determination of the color was calculated on the basis of the formula:

mm Pfund=−38.70+371.39×Abs,

where: *Abs*—is the absorbance value read [15].

#### 2.2.7. Determination of Radical Scavenging Capacity by DPPH Assay (DPPH)

The anti-DPPH radical scavenging ability was tested according to the method described by Sánchez-Moreno et al. [16] with own modification.

The honey samples were dissolved in distilled water to obtain a concentration of 1 g/mL. Then 1800 mL of methanol DPPH solution (concentration 0.04 mg/mL) was added to 200 mL of honey solutions, the absorbance was measured at 517 nm. The samples were incubated for 30 minutes in the dark at room temperature, after which time the absorbance was measured again using a spectrophotometer (Hitachi, Tokyo, Japan). The percentage of free radical scavenging was calculated from the formula:

$$\mathrm{DPPH}\;[\%]=[1-\frac{Ax}{A0}]\;\times \;100,$$

where *Ax* is the absorbance for the honey solution and *A*0 is the absorbance of the control (the honey solution before incubation).

#### 2.2.8. Determination of FRAP

The FRAP test was performed according to the methodology described by Benzie and Strain [17], with our own modifications.

The FRAP reagent was prepared which contained 2.5 mL of a 10 mM TPTZ solution in 40 mM HCl, 2.5 mL of 20 mM FeCl_3_, and 25 mL of 0.3 M acetate buffer, pH 3.6. On 96-well plates, 20 µL of honey solution was mixed with 180 µL of FRAP reagent. The plates were incubated for 10 min at 37 °C and the absorbance of the reaction mixture was measured with a plate reader (UVM 340, Biogenet, Józefów, Poland) at 593 nm. The results are expressed as equivalent µmoles of Fe^2+^/mL of sample.

### 2.3. Statistical Analysis

Statistical analyses of the obtained data were performed using the Statistica v.13.3 software (TIBCO Software Inc., Palo Alto, CA, USA). The normality of the data distribution was assessed by the Kolmogorov–Smirnov test, Lillefors test and the Shapiro–Wilk test. *P* values < 0.05 were considered significantly different.

The relationship between continuous categorized data was assessed by standard ANOVA. For better data classification and overall parameter evaluation, chemometric analyses were performed, including cluster analysis and principal component analysis.

## 3. Results

Table 2 presents the results of the melissopalinological analysis. It was shown that among honeys from Italy, only samples of chestnut honeys were all characterized by the correct variety definition. In fact, all acacia, dandelion, and raspberry honeys from Poland should be given a different name of the variety. Of the Spanish honeys, only the chestnut and lavender honeys were correctly named. Overall, only 62% of the honey was correctly labeled by beekeepers.

Table 3 presents the results for individual determinations, taking into account the division into varieties according to the declaration on the packaging.

Among honeys originating in Italy, chestnut honeys were characterized by the highest medians for most of the parameters tested: TPC (87.40 mg GAE/100 g), 123.1 mm Pfund, 64.4% free radical scavenging, and 0.216 uM of Fe^2+^/mL sample. The highest median color intensity was found for eucalyptus honeys (0.275 mAU) (Table 3).

The analysis of selected antioxidant parameters of Polish honeys showed that buckwheat honeys were characterized by the highest medians for most parameters: 213.05 mg GAE/100 g, 1.138 mAU, and 0.394 µM of Fe^2+^/mL sample. Among honeydew honeys, the highest color median was demonstrated for honeydew coniferous honeys (244.8 mm Pfund), and the highest free radical scavenging ability was found in honeydew deciduous honeys (68.5%) (Table 3).

The analysis of the antioxidant parameters of Spanish honeys did not show any unequivocal conclusions. Chestnut honeys were characterized by the highest median TPC (121.40 mg GAE/100 g), color intensity (1.021 mAU), while heath honeys were distinguished by a high color value on the Pfund scale (251.6 mm Pfund) and a high value in the FRAP test (0.348 uM of Fe^2+^/mL sample). Thyme honeys showed the highest free radical scavenging ability (79.7%) (Table 3).

Statistical analyses showed the greatest differences between the medians for individual parameters in the case of honeys from Poland and Italy, e.g., between buckwheat and lemon honeys (FRAP, CP, TPC), between Polish honeys such as buckwheat and acacia honeys (FRAP, CI, TPC), and between Italian and Spanish honeys such as lemon and chestnut honeys (FRAP, CP, TPC), lemon and heath honey (FRAP, CP, TPC), and lemon and thyme (FRAP, CP, TPC, DPPH) (Table 3).

Table 4 presents the results for individual parameters, the division into varieties was used according to the performed melissopalinological analysis. Chestnut honeys were characterized by the highest TPC value (87.40 mg GAE/100 g), color on the Pfund scale (123.1 mm Pfund) and the highest result in the FRAP test (0.216 uM of Fe^2+^/mL sample). The highest median color intensity (0.292 mAU) and the highest free radical scavenging ability (66.8%) were demonstrated for eucalyptus honeys.

Among the honeys obtained from Poland, buckwheat honeys were characterized by the highest median for three parameters: TPC (213.05 mg GAE/100 g), color intensity (1.138 mAU), and the value obtained in the FRAP assay (0.394 µM of Fe^2+^/mL sample). The highest median for the color parameter on the Pfund scale was characteristic for honeydew coniferous honeys (244.8 mm Pfund), and the highest free radical scavenging capacity was for nectar-honeydew honeys (68.6%).

Among the honeys that were purchased from Spain, in terms of antioxidant properties, the highest values for four out of five tested parameters were found for heath honeys: TPC (104.02 mg GAE/100 g), color intensity (0.465 mAU), color on the Pfund scale (243.3 mm Pfund), and the FRAP assay result (0.319 µM of Fe^2+^/mL sample). Thyme honey was characterized by the highest scavenging capacity of free radicals (80.2%).

Further chemometric analyses were performed on the basis of the appropriate varieties, shown in the melissopalinological method.

**Figure 1:**
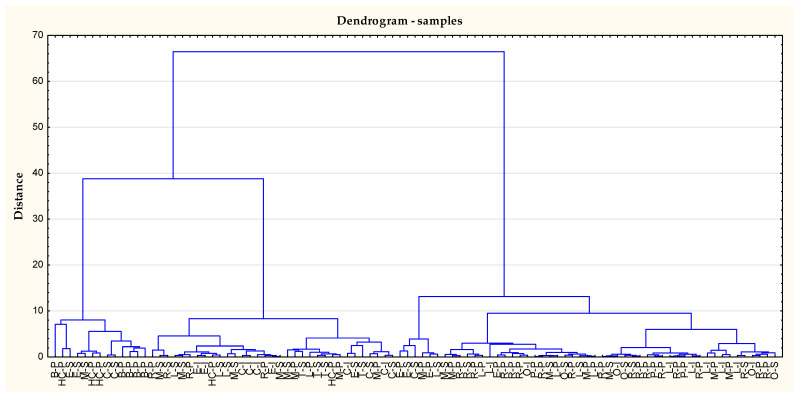
Dendrogram for the studied cases. B-P—buckwheat-Poland, C-I—chestnut-Italy, C-S—chestnut-Spain, E-I—eucalyptus-Italy, E-P—*Ericace*-Poland, E-S—*Erica-*Spain, HC-P—honeydew-Poland, L-I—*Lotus corniculatus* L.-Italy, L-P—lime-Poland, L-S—lavender-Spain, M-P—multifloral-Poland, M-P—multifloral-Poland, M-S—multifloral-Spain, O-I—orange-Italy, O-S—orange-Spain, P-P-phacelia-Poland, R-P—rape-Poland, R-S—rosemary-Spain, T-S—thyme-Spain.

On the basis of performed the cluster analysis we can observe that first cluster was mainly dark honeys, such as buckwheat, honeydew coniferous, or multifloral (Figure 1).

Principal component analysis distinguished three main components. The first component accounted for 65.82% of the total variance, the second—21.89%, and the third—6.84%, which was a total of 94.55%.

**Figure 2:**
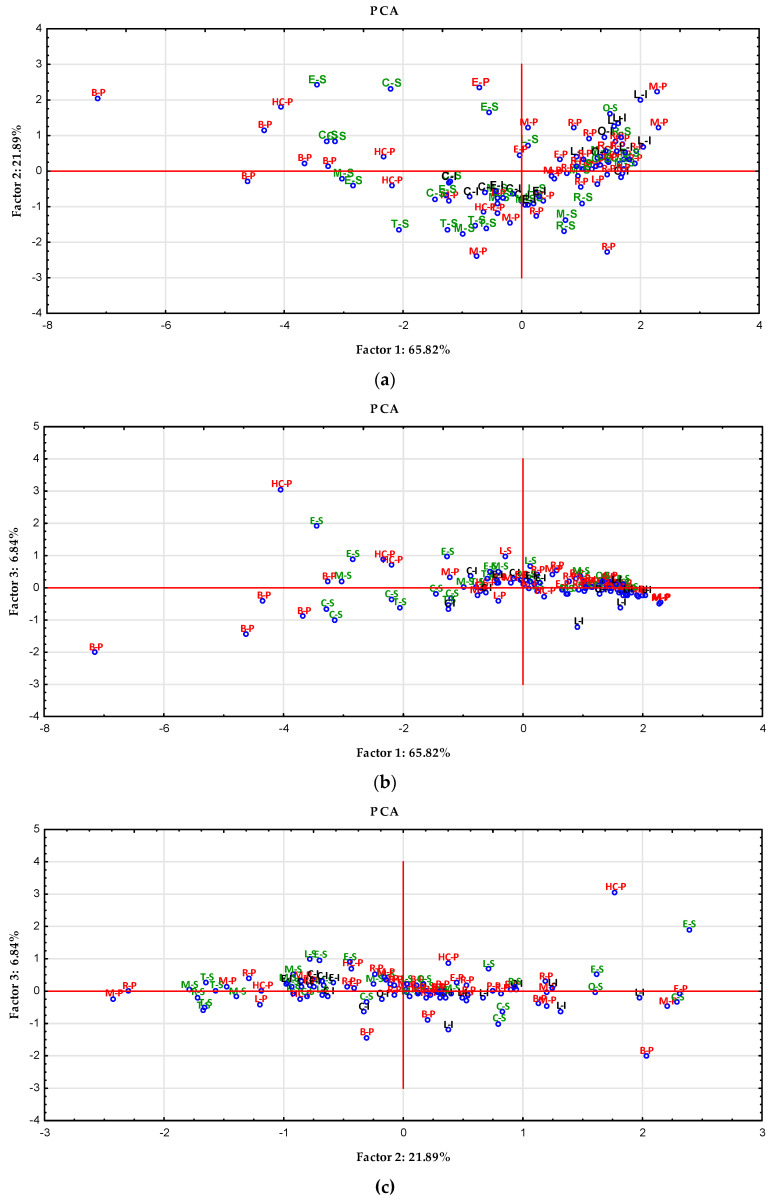
Projection of cases depending on the selected antioxidants parameters in a two-factor plane: factor 1 × factor 2 (**a**), factor 1 × factor 3 (**b**), factor 2 × factor 3 (**c**).

Figure 2 presents a scatter plot of the assessed bee honey varieties in the space of two main components.

DPPH showed practically no correlation with the first component (0.01), the correlation of the remaining four variables is negative, at a similar level (FRAP: −0.51, TPC: −0.52, CI: −0.49, Pfund: −0,48). In the case of the second component, the strongest negative correlation was demonstrated with DPPH (−0.95), negative correlation was also shown by FRAP (−0.24), and TPC—practically no correlation (−0.06). The correlation with CI (0.14) and Pfund (0.15) is at a similar level. In the case of the third factor, the strongest positive correlation is seen with the Pfund factor (0.74). There is also a positive correlation between FRAP (0.16). No correlation (0.01) for DPPH. A stronger negative correlation was demonstrated for CI (−0.59) than for TPC (−0.28).

The comparison of antioxidant properties between honeys from Poland, Italy, and Spain shows no differences between these parameters—except for a significantly higher median color on the Pfund scale of honeys from Spain, compared to honeys from Italy (Table 5).

## 4. Discussion

The principle of determining the variety of honey is the method based on the assessment of the percentage of pollen grains of various plant species present in the honey sediment. The variety of honey is given the name of the plant on the basis of whichever grains are predominantly present [12]. The performance of these determinations is necessary to qualify the honey samples to the varieties, and therefore constitutes the basis for inference about properties, including antioxidant properties. Incorrect labeling may mislead consumers, and in the case of scientific research, result in incorrect formulation of conclusions as to the properties of the varieties. Before starting the analyses, many authors obtain certificates of the authenticity of the variety from beekeepers [18]. At the same time, there are many varieties on sale, the names of which sometimes constitute an incentive to purchase rather than an indication of the actual variety.

The antioxidant properties of bee honeys are one of the compelling reasons why consumers or patients buy these bee products. There are many methods of determining the antioxidant properties of honey—the most common of them are: TPC, DPPH, and FRAP. Color intensity and color on the Pfund scale are parameters that may correlate with antioxidant properties.

TPC is one of the most frequently performed assays to evaluate the total content of phenolic compounds in food samples. Research conducted on buckwheat honeys from Poland by Haladarga et al. (2020) [18] showed almost 10 times lower TPC value than our analyses: 22.33 ± 0.81 and 17.95 ± 1.57 mg GAE/100 g vs. 212.63 ± 37.71 mg GAE/100 g. It should be emphasized that despite this fact, buckwheat honeys assessed by the above authors were characterized by the highest value of the tested parameter.

The DPPH test is based on the measurement of the free radical scavenging activity of a sample. This is observed as a decrease in the absorbance of the methanolic DPPH solution at 517 nm [19]. The research conducted by Wilczyńska et al. (2010) [20] was aimed at assessing the antioxidant properties of honeys from Poland. One of the parameters assessed was the ability to scavenge free radicals. Out of three samples of buckwheat honeys, one honey showed 100% free radical scavenging. Two more samples showed the values 68.88% and 56.39%, respectively. The honeys tested in our publication showed the ability to scavenge free radicals from 21.1% to 57.9%. Surprisingly, 100% free radical scavenging capacity was demonstrated in the case of multiflorous, phacelia, and rapeseed honeys.

One of the important parameters for the assessment of antioxidant properties is FRAP. This test is based on the direct measurement of antioxidants in a sample. It is based on the ability of the sample to reduce Fe^3+^/Fe^2+^ [19]. The reduction power of three varieties of honey was studied by Alzahrani et al. (2012) [21]. They showed that acacia honey has the highest potency (1.366 ± 0.006), and that it was higher than manuka honey (1.2106 ± 0.005). Our research also covered acacia honey from Poland—the name of the variety was based on the declaration of beekeepers. Interestingly, honeys of this variety showed approximately 15 times lower reduction power than buckwheat honeys (Table 2).

One way to determine color is the Pfund scale. The term color on the Pfund scale was introduced to classify color on a numerical scale or as categories. The latter method is based on assigning numerical results to each class: white water (0–8), extra white (9–17), white (18–34), extra light amber (35–50), light amber (51–85), amber (86–114), and dark amber (over 115) [15]. As these terms do not correspond directly to the nomenclature of honeys in Poland, we used universal numerical values in this publication. Data in the literature indicate a low color value in the case of acacia honeys (7.01 ± 8.27). Lime (71.79 ± 31.98) and rapeseed (86.91 ± 51.06) honeys are characterized by a much higher value of color. The color of honeydew honeys is much higher—at the level of 131.16 ± 51.64 mm Pfund [22]. Our analyses showed a slightly lower value for linden (61.1 ± 11.5) and rapeseed (53.6 ± 16.7) honeys. The color of honeydew honeys was the highest (244.8 ± 171.8), while buckwheat honeys (248.2 ± 64 mm Pfund) were characterized by slightly higher.

Another way to objectively assess the color of honey is to define it as the color intensity. The research conducted for buckwheat honeys by Beretta et al. (2006) [14] showed approximately 1.5 times higher color intensity than our research (2245 vs. 1421 mAU).

The antioxidant properties of bee honeys are the subject of research by many authors, due to the possibility of using their prophylactic properties in various disease entities. For example, Kishore et al. (2011) [23] compared the antioxidant properties of Tualang and other honeys in order to emphasize the beneficial properties of the former variety. Interestingly, the authors showed TPC for this variety at the level of 83.96 ± 4.53 mg GAE/100 g, while FRAP at the level of 121.89 ± 3.87 µM Fe^2+^/100 g. Buckwheat from Poland is characterized by almost three times higher total content of phenolic compounds (212.63 ± 5.97 mg GAE/100 g) and almost three times higher value obtained in the FRAP test (0.394 µM Fe^2+^/mL sample).

Buckwheat honeys are characterized by specific antioxidant properties. Interesting results were published by Deng et al. (2018). The authors compared the quality of buckwheat honeys from China and manuka honeys, the price of which is several times higher than that of other honey varieties. Buckwheat honey, as in our publication, was characterized by a high content of phenolic compounds (149.8 ± 3.7 mg GAE/100 g)—higher than manuka honey (56.1 ± 0.3 mg GAE/100 g) [24].

The influence of buckwheat honey consumption on antioxidant parameters in blood serum was assessed, among others, by Gheldof et al. (2003) [25]. Men (*n* = 25) consumed: 500 mL of water (control), water with buckwheat honey (160 g/L), black tea, black tea with buckwheat honey (160 g/L), black tea with sugar analogue (160 g/L, 45% fructose + 35% glucose + 20% water). The antioxidant capacity of the serum was assessed using the ORAC test (the oxygen radical absorbance capacity)—ability to absorb oxygen radicals, the TBARS test (the thiobarbituric acid reactive substances), and the ex vivo susceptibility test of serum lipoprotein to oxidation induced by Cu^2+^. The authors demonstrated the effectiveness of consuming buckwheat honey in increasing the antioxidant capacity in the ORAC test by 7% (*p* < 0.05).

Data in the literature indicate a high correlation between the parameters determining the antioxidant properties of natural honey. For example, a very high positive correlation was found between TPC and FRAP (R = 0.89), as well as between TPC and DPPH (R = 0.92) [14]. 

PCA was used as a method of showing similarities for honey samples from Algeria. One of the parameters tested was the content of polyphenols. Nine principal components were obtained, explaining 72% of the variance. The authors obtained a grouping of honey samples from botanical origin, among others [24]. Our analyses, however, distinguished three main components, which explained as much as 94.55% of the variability.

Buckwheat honey is valued by consumers for its organoleptic properties, distinct taste, and aroma. Buckwheat honeys from Poland are characterized by a high proportion of main pollen grains, compared to buckwheat honeys from other countries. In this bee honey, various derivatives of phenolic compounds have been demonstrated: benzoic acid derivatives (protocatechuic acid and p-hydroxybenzoic acid), cinnamic acid derivatives (3-O-ceffeoylquinic acid, 5-O-caffeoylquinic acid, coumaroyl hexoside, caffeic acid, 5-O-p-coumaroylquinic acid, p-coumaric acid, cinnamic acid, ferruli acid, benzyl caffeate, preny caffeate, cinnamyl caffeate), flavones (luteolin 6-C-hexoside, luteolin 8-C-hexoside, vitexin, luteolin, apigenin, chrysoeriol, tricin, chrysin, acacetin), flavanols (quercetin 3-O-(6″-rhamnosyl)-hexoside, quercetin 3-O-galactoside, quercetin 3-O-rhamnoside, quercetin, quercetin 3-methyl ether, kaempferol, herbacetin 8-methyl ether, isorhamnetin, dimethyl quercetin, rhamnetin, galangin, kaempferide), flavanols (aromodedrin, pinobanksin 5-methyl ether, pinobanksin, pinobanksin 3-acetate, pinobanksin 3-butyrate, pinobanksin 3-pentanoate), and flavanone (pinocembrin) [26].

Our study has several limitations. We obtained various amounts of samples from individual countries. Future research should include the same number of samples from each variety and represent all the varieties sourced in a given country, in order to reach unambiguous conclusions. We did not have information from beekeepers as to the period of obtaining individual varieties. An important aspect that should be taken into account is the annual honey harvesting period, as the antioxidant parameters may change, even within a given variety, over the course of months. In addition, in our study, we considered several of the most popular methods of assessing antioxidant properties. In future research, the assessment of the properties of bee honeys should be extended to include ABTS test, and total flavonoids.

## 5. Conclusions

In summary, the analysis of antioxidant properties, taking into account the country of origin, did not show any differences in antioxidant properties between honeys obtained from Poland, Italy, and Spain. Origin has no effect on antioxidant capacity—the most important thing is the source of the nectar or honeydew from which the bee honey is made. The evaluation of the antioxidant properties of individual varieties showed that buckwheat honeys, which are botanically confirmed, are characterized by a high total content of phenolic compounds and a high ability to reduce iron compounds, confirmed in the FRAP test. Moreover, their color intensity, assessed spectrophotometrically, is the highest, compared to other honeys. These results can be the basis for the promotion of honey as a source of antioxidants.

## Figures and Tables

**Table 1:** Characteristics of the analyzed honeys—breakdown in accordance with the manufacturers’ declaration.

The Origin of the Samples	Variety of Samples
Italy (*n* = 20)	chestnut (*n* = 5), eucalyptus (*n* = 5), lemon (*n* = 5), orange (*n* = 5)
Poland (*n* = 50)	acacia (*n* = 5), buckwheat (*n* = 5), dandelion (*n* = 5), heather (*n* = 5), honeydew coniferous (*n* = 5), honeydew deciduous (*n* = 5), linden (*n* = 5), phacelia (*n* = 5), rape (*n* = 5), raspberry (*n* = 5)
Spain (*n* = 35)	almond (*n* = 5), chestnut (*n* = 5), heath (*n* = 5), lavender (*n* = 5), orange (*n* = 5), rosemary (*n* = 5), thyme (*n* = 5)

**Table 2:** Results of the melissopalinological analysis.

Varieties Declared by Beekeepers	The Origin of the Samples	Percentage of Correctly Classified Samples	Percentage of Incorrectly Classified Samples
chestnut	Italy	100	0
eucalyptus		60	40
lemon		20	80
orange		40	60
acacia	Poland	0	100
buckwheat		100	0
dandelion		0	100
heather		60	40
honeydew coniferous		100	0
honeydew deciduous		40	60
linden		80	20
phacelia		60	40
rape		100	0
raspberry		0	100
almond	Spain	20	80
chestnut		100	0
heath		80	20
lavender		100	0
orange		60	40
rosemary		80	20
thyme		80	20
TOTAL		62	38

**Table 3:** The results of research on antioxidant properties—division into varieties according to the manufacturers’ declaration.

Varieties Declared by Beekeepers (Sign)	Total Phenolic Content	Color Intensity	Color in Pfund Scale	DPPH	FRAP (Equivalent µM of Fe^2+^/mL of Sample)
	(mg GAE/100 g)	(mAU)	(mm Pfund)	(%)	
**Av. ± SD** **Min–Max** **Med.** **Q1–Q3**					
**THE ORIGIN OF THE SAMPLES: ITALY**					
chestnut (A)	95.1 ± 16.7	0.346 ± 0.241	121.8 ± 12.98	63.5 ± 3.4	0.222 ± 0.030
	78.58–114.00	0.156–0.694	104.7–140.4	58.1–67.4	0.193–0.265
	87.4	0.192	123.1	64.4	0.216
	83.38–112.20	0.185–0.506	116.6–124.4	63.0–64.8	0.197–0.237
eucalyptus (B)	53.53 ± 10.6	0.263 ± 0.081	103.2 ± 24.5	60.6 ± 10.8	0.170 ± 0.048
	47.64–72.41	0.176–0.376	80.6–142.2	43.2–71.0	0.118–0.214
	48.91	0.275	100.4	64.1	0.188
	47.99–50.73	0.194–0.292	84.8–107.8	57.7–66.8	0.119–0.210
lemon (C)	21.47 ± 2.94	0.134 ± 0.111	44.5 ± 21.7	31.5 ± 10.2	0.014 ± 0.008
	18.01–24.34	0.051–0.323	31.7–83.1	15.2–42.1	0.006–0.0248
	21.89	0.12	35.1	32	0.011
	18.90–24.23	0.054–0.126	35.1–37.3	30.8–37.6	0.008–0.019
orange (D)	29.84 ± 5.41	0.222 ± 0.310	43.1 ± 15.7	46.4 ± 8.3	0.066 ± 0.027
	22.89–36.04	0.080–0.776	33.5–71.1	35.4–57.4	0.047–0.113
	29.58	0.08	37	44.2	0.06
	26.49–34.21	0.075–0.108	35.8–38.1	44.2–51.5	0.051–0.059
TOTAL	49.99 ± 30.83	0.241 ± 0.206	78.1 ± 40.0	50.5 ± 15.3	0.118 ± 0.089
	18.01–114.00	0.050–0.776	31.7–142.2	15.2–71.0	0.006–0.265
	41.84	0.18	81.8	54.4	0.115
	24.28–75.49	0.094–0.307	36.4–112.2	39.9–64.2	0.036–0.204
**THE ORIGIN OF THE SAMPLES: POLAND**					
acacia (E)	21.29 ± 5.97	0.043 ± 0.014	21.9 ± 21.7	34.2 ± 16.0	0.026 ± 0.023
	14.70–28.15	0.025–0.050	0.1–49.8	9.3–51.0	0.004–0.061
	24.04	0.045	25.3	37.1	0.026 *** E/F
	15.31–24.26	0.033–0.052	0.1–34.0	30.0–43.6	0.006–0.031
buckwheat (F)	212.63 ± 37.71	1.421 ± 0.724	248.2 ± 63.98	39.1 ± 15.0	0.391 ± 0.014
	167.75–261.05	0.770–2.605	182.8–351.1	21.1–57.9	0.370–0.407
	213.05 *** C/F, ** D/F, *** E/F	1.138 *** E/F	237.3 ** C/F	43.5	0.394 *** C/F, * D/F
	184.95–236.35	1.015–1.578	212.2–257.8	26.6–46.3	0.383–0.400
dandelion (G)	46.84 ± 13.85	0.206 ± 0.118	133.8 ± 47.6	49.0 ± 16.0	0.125 ± 0.059
	34.70–68.85	0.111–0.379	87.4–211.3	30.1–67.1	0.090–0.229
	40.75	0.133	127.9	53.1	0.1
	38.23–51.71	0.130–0.279	104.7–137.6	34.7–59.9	0.098–0.108
heather (H)	82.32 ± 25.65	0.353 ± 0.089	122.6 ± 22.9	46.0 ± 34.3	0.152 ± 0.050
	49.42–116.60	0.265–0.471	90.2–149.6	6.8–100.0	0.118–0.238
	76.48	0.351	120.9	45	0.141
	71.72–97.37	0.271–0.406	113.9–138.3	30.3–47.9	0.120–0.142
honeydew coniferous (I)	98.38 ± 23.03	0.517 ± 0.151	244.8 ± 171.8	54.8 ± 20.5	0.276 ± 0.079
	64.57–120.75	0.309–0.643	77.9–518.8	24.4–74.2	0.152–0.343
	109.35	0.61	244.8	61.1	0.312
	85.25–112.00	0.404–0.622	122.3–259.4	44.4–69.7	0.243–0.328
honeydew deciduous (J)	76.42 ± 17.98	0.402 ± 0.157	150.0 ± 29.3	67.3 ± 5.0	0.189 ± 0.052
	45.58–92.41	0.237–0.559	126.4–200.1	59.3–71.5	0.107–0.242
	82.60 * C/J	0.424 * E/J	138.2	68.5	0.199 * C/J
	78.42–83.08	0.244–0.549	134.8–150.4	65.8–71.3	0.178–0.219
linden (K)	51.80 ± 21.79	0.186 ± 0.169	78.4 ± 14.3	59.6 ± 10.5	0.100 ± 0.049
	36.61–89.66	0.098–0.489	61.0–100.8	51.0–76.7	0.074–0.188
	42.02	0.109	76.4	53.9	0.077
	40.04–50.70	0.108–0.128	75.7–78.7	53.8–62.9	0.077–0.082
phacelia (L)	42.97 ± 28.67	0.158 ± 0.207	76.3 ± 61.3	61.5 ± 24.3	0.098 ± 0.084
	25.17–93.74	0.050–0.528	32.9–184.5	39.6–100.0	0.042–0.44
	30.96	0.06	55.6 *** C/L, ** G/L	50.2	0.057
	28.62–36.39	0.058–0.095	51.4–56.9	47.3–70.2	0.051–0.094
rape (M)	30.61 ± 3.24	0.064 ± 0.014	62.0 ± 17.6	49.9 ± 7.1	0.056 ± 0.012
	27.28–35.96	0.050–0.080	44.4–85.3	43.0–58.1	0.043–0.075
	29.88 * F/M	0.063 ** F/M	61.7	48.7	0.056 ** F/M
	29.26–30.69	0.051–0.078	45.6–72.9	43.3–56.2	0.046–0.058
raspberry (N)	48.72 ± 16.14	0.162 ± 0.061	91.6 ± 23.6	63.66 ± 15.33	0.164 ± 0.050
	34.13–73.20	0.107–0.246	70.1–126.7	45.95–79.39	0.118–0.240
	40.82	0.14	79.4	62.25	0.166
	38.64–56.85	0.113–0.204	77.2–104.7	51.74–78.97	0.121–0.173
TOTAL	71.20 ± 56.32	0.351 ± 0.453	122.9 ± 92.8	52.5 ± 19.5	0.157 ± 0.114
	14.70–261.05	0.025–2.605	0.1–518.8	6.8–100.0	0.004–0.407
	50.06	0.22	102.7	51.4	0.119
	34.13–89.66	0.080–0.471	61.7–149.6	43.3–65.8	0.074–0.238
**THE ORIGIN OF THE SAMPLES: SPAIN**					
almond (O)	68.14 ± 13.89	0.290 ± 0.113	138.4 ± 22.7	67.0 ± 6.57	0.149 ± 0.050
	44.06–77.78	0.117–0.381	116.8–176.6	59.3–74.3	0.067–0.206
	73.98	0.336	131.5	65.8	0.158
	68.79–76.13	0.238–0.377	129.8–137.3	62.6–73.2	0.152–0.159
chestnut (P)	116.06 ± 14.67	1.043 ± 0.383	186.5 ± 43.1	43.0 ± 21.7	0.275 ± 0.043
	100.14–129.95	0.665–1.441	136.8–236.7	10.5–66.9	0.218–0.317
	121.40 ** C/P, ** E/P	1.021 * C/P, *** E/P, ** M/P	198.4	40.3	0.288 * C/P, * D/P, * E/P
	100.61–128.20	0.667–1.423	147.1–213.5	39.2–58.3	0.243–0.308
heath (Q)	111.06 ± 17.93	0.563 ± 0.460	255.96 ± 101.71	41.1 ± 27.1	0.320 ± 0.052
	88.59–131.25	0.045–0.957	137.8–403.69	8.8–63.5	0.244–0.367
	105.84 ** C/Q, * E/Q	0.851	251.6 * C/Q	57.73	0.348 ** C/Q, ** E/Q
	102.20–127.40	0.079–0.886	192.1–294.6	14.3–61.2	0.290–0.351
lavender (R)	53.23 ± 7.95	0.238 ± 0.111	130.3 ± 39.8	60.4 ± 13.9	0.154 ± 0.041
	40.65–62.57	0.116–0.408	92.2–187.6	39.0–71.9	0.092–0.207
	54.73	0.213	113.1	68.1	0.159
	52.81–55.38	0.176–0.276	103.4–154.7	53.8–69.3	0.148–0.162
orange (S)	30.57 ± 3.44	0.089 ± 0.027	62.8 ± 14.9	45.0 ± 13.2	0.053 ± 0.017
	27.84–34.48	0.071–0.136	42.2–84.2	21.9–53.6	0.034–0.071
	28.42 *** F/S	0.076 * F/S	62.5	51.5	0.049 ** F/S
	27.93–34.17	0.074–0.087	62.1–63.2	46.3–51.8	0.042–0.071
rosemary (T)	41.06 ± 17.00	0.117 ± 0.052	57.6 ± 13.8	64.3 ± 20.3	0.098 ± 0.068
	23.22–59.64	0.051–0.179	40.7–78.3	38.2–84.9	0.015–0.165
	43.35	0.138	58.9	71	0.124 ** L/T, * Q/T
	24.01–55.10	0.078–0.142	50.8–59.5	47.9–79.4	0.015–0.165
thyme (U)	116.6 ± 42.4	0.359 ± 0.192	104.0 ± 11.9	79.3 ± 2.4	0.316 ± 0.038
	92.2–190.8	0.195–0.673	90.7–118.2	75.6–81.8	0.276–0.358
	94.3 * C/U, * E/U	0.356	100.4 ** C/U	79.7 * C/U, * E/U	0.314 ** C/U, ** E/U
	92.66–112.95	0.210–0.364	96.1–114.5	78.8–80.7	0.281–0.352
TOTAL	76.67 ± 39.58	0.386 ± 0.382	133.6 ± 78.0	57.2 ± 20.6	0.195 ± 0.110
	23.22–190.80	0.045–1.441	40.7–403.7	8.8–84.9	0.015–0.367
	73.98	0.213	116.8	61.2	0.165
	43.35–102.20	0.116–0.665	78.3–176.6	46.3–73.2	0.092–0.290

* *p* < 0.05, ** *p* < 0.01, *** *p* < 0.001, DPPH—method using 2,2-diphenyl-1-picryl-hydrazyl-hydrate.

**Table 4:** The results of research on antioxidant properties—division into varieties according to the results of melissopalinological analysis.

Varieties According to the Melissopalinological Analysis (Sign)	Total Phenolic Content	Color Intensity	Color in Pfund Scale	DPPH	FRAP (Equivalent µmoles of Fe^2+^/mL of Sample)
	(mg GAE/100 g)	(mAU)	(mm Pfund)	(%)	
**Av. ± SD** **Min–Max** **Med.** **Q1–Q3**					
**THE ORIGIN OF THE SAMPLES: ITALY**					
chestnut, *n* = 5 (A)	95.1 ± 16.7	0.346 ± 0.241	121.8 ± 12.98	63.5 ± 3.4	0.222 ± 0.030
	78.58–114.00	0156–0.694	104.7–140.4	58.1–67.4	0.193–0.265
	87.4	0.192	123.1	64.4	0.216
	83.38–112.20	0.185–0.506	116.6–124.4	63.0–64.8	0.197–0.237
eucalyptus, *n* = 3 (B)	56.01 ± 14.20	0.314 ± 0.054	116.8 ± 22.3	67.3 ± 3.4	0.204 ± 0.013
	47.64–72.41	0.275–0.376	100.4–142.2	64.1–71.0	0.188–0.214
	47.99	0.292	107.8	66.8	0.21
	47.64–72.41	0.275–0.376	100.4–142.2	64.1–71.0	0.188–0.214
lemon, *n* = 1 (C)	21.89	0.12	35.1	37.6	0.025
	-	-	-	-	-
	21.89	0.12	35.1	37.6	0.025
	-	-	-	-	-
*Lotus corniculatus* L., *n* = 6 (D)	22.47 ± 3.33	0.233 ± 0.285	43.3 ± 19.6	36.0 ± 12.9	0.024 ± 0.020
	18.01–24.49	0.051–0.776	31.7–83.1	15.2–51.5	0.006–0.051
	23.56 *** A/D	0.099	36.4	37	0.015
	18.90–24.24	0.054–0.230	35.1–37.3	30.8–44.2	0.008–0.047
multifloral, *n* = 1 (E)	50.73	0.176	84.8	43.2	0.119
	-	-	-	-	-
	50.73	0.176	84.8	43.2	0.119
	-	-	-	-	-
orange, *n* = 3 (F)	33.28 ± 3.33	0.088 ± 0.018	47.5 ± 20.5	45.6 ± 11.0	0.077 ± 0.031
	29.58–36.04	0.075–0.108	33.5–71.1	35.4–57.3	0.006–0.113
	34.21 * A/F	0.080 * A/F	38.1	44.2	0.059
	29.58–36.04	0.075–0.108	33.5–71.1	35.4–57.3	0.006–0.113
other, *n* = 1 (G)	48.91	0.194	80.6	57.7	0.118
	-	-	-	-	-
	48.91	0.194	80.6	57.7	0.118
	-	-	-	-	-
TOTAL	49.99 ± 30.83	0.241 ± 0.206	78.1 ± 40.0	50.5 ± 15.3	0.118 ± 0.089
	18.01–114.00	0.050–0.776	31.7–142.2	15.2–71.0	0.006–0.265
	41.84	0.18	81.8	54.4	0.115
	24.28–75.49	0.094–0.307	36.4–112.2	39.9–64.2	0.036–0.204
**THE ORIGIN OF THE SAMPLES: POLAND**					
buckwheat, *n* = 5 (H)	212.63 ± 37.71	1.421 ± 0.724	248.2 ± 64	39.1 ± 15.0	0.391 ± 0.014
	167.75–261.05	0.770–2.605	182.8–351.1	21.1–57.9	0.370–0.407
	213.05 *** D/H, ** F/H	1.138 ** D/H, ** F/H	237.3	43.5	0.394 *** D/H, ** F/H
	184.95–236.35	1.015–1.578	212.2–257.8	26.6–46.3	0.383–0.400
heather, *n* = 3 (I)	80.83 ± 33.80	0.314 ± 0.080	126.0 ± 31.6	33.3 ± 22.9	0.133 ± 0.014
	49.42–116.60	0.265–0.407	90.2–149.6	6.8–47.9	0.118–0.142
	76.48	0.271	138.3	45	0.141 ** D/I
	49.42–116.60	0.265–0.406	90.2–149.6	6.8–47.9	0.118–0.142
honeydew coniferous, *n* = 5 (J)	98.38 ± 23.03	0.517 ± 0.151	244.8 ± 171.8	54.8 ± 20.5	0.276 ± 0.079
	64.57–120.75	0.309–0.643	77.9–518.8	24.4–74.2	0.152–0.343
	109.35 * D/J	0.61	244.8	61.1	0.312 * D/J
	85.25–112.00	0.404–0.622	122.3–259.4	44.4–69.7	0.243–0.328
honeydew deciduous, *n* = 2 (K)	87.50 ± 6.94	0.486 ± 0.089	163.2 ± 52.1	70.0 ± 2.2	0.221 ± 0.031
	82.60–92.41	0.424–0.549	126.4–200.1	68.5–71.5	0.199–0.242
	87.5	0.486	163.2	70	0.221
	82.60–92.41	0.424–0.549	126.4–200.1	68.5–71.5	0.199–0.242
linden, *n* = 4 (L)	54.25 ± 24.35	0.206 ± 0.189	78.9 ± 16.4	61.1 ± 11.5	0.105 ± 0.055
	36.61–89.66	0.098–0.489	61.0–100.8	51.0–76.7	0.074–0.188
	45.37	0.118	77	58.4	0.079
	38.32–70.18	0.103–0.308	68.3–89.5	52.4–69.8	0.075–0.135
multiflower, *n* = 8 (M)	56.70 ± 32.38	0.250 ± 0.190	92.3 ± 64.7	54.0 ± 29.7	0.130 ± 0.101
	14.70–93.37	0.025–0.528	0.1–184.5	9.3–100.0	0.004–0.244
	58.65	0.242	109.3	56.6	0.113
	28.67–83.47.39	0.070–0.411	38.3–129.5	30.1–74.6	0.042–0.239
nectar-honeydew, *n* = 2 (N)	80.75 ± 3.30	0.401 ± 0.222	142.6 ± 11.0	68.6 ± 3.9	0.198 ± 0.029
	78.42–83.08	0.244–0.559	134.8–150.4	65.8–71.3	0.178–0.219
	80.75	0.401	142.6	68.6	0.198
	78.42–83.08	0.244–0.559	134.8–150.4	65.8–71.3	0.178–0.219
phacelia, n = 3 (O)	42.97 ± 28.67	0.158 ± 0.207	76.3 ± 61.3	61.5 ± 24.3	0.098 ± 0.084
	25.17–93.74	0.050–0.528	32.9–184.5	39.6–100.0	0.042–0.44
	30.96	0.060 * H/O	55.6 * H/O	50.2	0.057 * H/O
	28.62–36.39	0.058–0.095	51.4–56.9	47.3–70.2	0.051–0.094
rape, *n* = 18 (P)	36.95 ± 11.79	0.118 ± 0.089	75.1 ± 32.5	53.6 ± 16.7	0.094 ± 0.054
	24.04–68.85	0.045–0.379	25.3–137.6	30.1–100.0	0.026–0.229
	34.41 ** A/P, *** H/P	0.094 ** A/P, * H/P	72	51.4	0.092 * A/P, ** H/P
	29.26–40.75	0.060–0.133	49.8–87.4	43.3–59.9	0.056–0.118
TOTAL	71.20 ± 56.32	0.351 ± 0.453	122.9 ± 92.8	52.5 ± 19.5	0.157 ± 0.114
	14.70–261.05	0.025–2.605	0.1–518.8	6.8–100.0	0.004–0.407
	50.06	0.22	102.7	51.4	0.119
	34.13–89.66	0.080–0.471	61.7–149.6	43.3–65.8	0.074–0.238
**THE ORIGIN OF THE SAMPLES: SPAIN**					
almond, *n* = 1 (Q)	68.8	0.377	131.5	62.6	0.159
	-	-	-	-	-
	68.8	0.377	131.5	62.6	0.159
	-	-	-	-	-
chestnut, *n* = 5 (R)	68.14 ± 13.89	0.290 ± 0.113	138.4 ± 22.7	67.0 ± 6.57	0.149 ± 0.050
	44.06–77.78	0.117–0.381	116.8–176.6	59.3–74.3	0.067–0.206
	73.98	0.336	131.5	65.8	0.158
	68.79–76.13	0.238–0.377	129.8–137.3	62.6–73.2	0.152–0.159
heath, *n* = 4 (S)	106.01 ± 16.08	0.465 ± 0.466	257.0 ± 117.4	37.0 ± 29.4	0.312 ± 0.056
	88.59–127.40	0.045–0.886	137.8–403.7	8.8–63.5	0.244–0.367
	104.02 * D/S	0.465	243.3 * D/S	37.7	0.319
	95.40–116.52	0.062–0.868	164.9–349.1	11.6–62.4	0.267–0.357
lavender, *n* = 5 (T)	53.23 ± 7.95	0.238 ± 0.111	130.3 ± 39.8	60.4 ± 13.9	0.154 ± 0.041
	40.65–62.57	0.116–0.408	92.2–187.6	39.0–71.9	0.092–0.207
	54.73	0.213	113.1	68.1	0.159
	52.81–55.38	0.176–0.276	103.4–154.7	53.8–69.3	0.148–0.162
multifloral, *n* = 9 (U)	70.39 ± 34.72	0.290 ± 0.269	118.6 ± 65.9	65.4 ± 11.8	0.172 ± 0.115
	27.84–131.25	0.076–0.957	42.2–251.6	46.3–79.4	0.041–0.352
	73.98	0.195	116.8	65.8	0.152
	44.06–77.78	0.136–0.336	63.2–137.3	57.7–74.3	0.071–0.206
orange, *n* = 3 (V)	30.17 ± 3.47	0.077 ± 0.009	69.6 ± 12.7	41.8 ± 17.2	0.051 ± 0.018
	27.93–34.17	0.071–0.087	62.2–84.2	21.9–51.8	0.034–0.071
	28.42	0.074 ** V/P	62.5	51.5	0.049
	27.93–34.17	0.071–0.087	62.1–84.2	21.9–51.8	0.034–0.071
rosemary, *n* = 4 (W)	37.55 ± 17.42	0.102 ± 0.045	57.3 ± 16.0	60.49 ± 21.3	0.086 ± 0.070
	23.22–59.64	0.051–0.142	40.7–78.3	38.2–84.9	0.015–0.165
	33.68	0.108	55.1	59.5	0.081
	23.62–51.49	0.064–0.140	45.7–68.9	43.1–77.9	0.027–0.144
thyme, *n* = 4 (X)	117.49 ± 48.88	0.400 ± 0.195	107.3 ± 10.7	79.4 ± 2.7	0.307 ± 0.038
	92.21–190.80	0.210–0.673	96.1–118.2	75.6–81.8	0.276–0.358
	93.48 * D/X	0.36	107.5	80.2 * D/X	0.298 ** D/X
	92.43–142.55	0.283–0.518	98.3–116.4	77.7–81.2	0.279–0.336
TOTAL	76.67 ± 39.58	0.386 ± 0.382	133.6 ± 78.0	57.2 ± 20.6	0.195 ± 0.110
	23.22–190.80	0.045–1.441	40.7–403.7	8.8–84.9	0.015–0.367
	73.98	0.213	116.8	61.2	0.165
	43.35–102.20	0.116–0.665	78.3–176.6	46.3–73.2	0.092–0.290

* *p* < 0.05, ** *p* < 0.01, *** *p* < 0.001, DPPH—method using 2,2-diphenyl-1-picryl-hydrazyl-hydrate.

**Table 5:** The results of research on antioxidant properties—division into country of origin.

Origin	Total Phenolic Content	Color Intensity	Color in Pfund Scale	DPPH	FRAP (Equivalent µM of Fe^2+^/mL of Sample)
(Sign)	(mg GAE/100 g)	(mAU)	(mm Pfund)	(%)	
Italy (A)	51.66 ± 32.81	0.255 ± 0.221	80.1 ± 42.2	51.3 ± 16.1	0.123 ± 0.094
	18.01–114.0	0.051–0,776	31.7–142.2	15.2–71.0	0.006–0.265
	36.04	0.184	83.1	57.3	0.113
	24.34–78.58	0.080–0.323	37.0–116.6	42.1–64.4	0.047–0.210
Poland (B)	70.08 ± 58.61	0.343 ± 0.470	117.3 ± 95.3	51.0 ± 19.7	0.153 ± 0.118
	14.70–261.05	0.025–2.605	0.1–518.8	6.8–100.0	0.004–0.407
	43.8	0.136	86.4	50.6	0.113
	30.96–89.66	0.078–0.406	61.0–138.2	43.0–61.1	0.061–0.238
Spain (C)	76.90 ± 40.15	0.386 ± 0.388	133.7 ± 79.1	57.1 ± 20.9	0.196 ± 0.111
	23.22–190.00	0.045–1.441	40.7–403.7	8.8–84.9	0.015–0.367
	75.05	0.211	115.7 * A/C	60.3	0.186
	43.35–102.20	0.116–0.665	78.3–176.6	46.3–73.2	0.092–0.290

* *p* < 0.05, DPPH—method using 2,2-diphenyl-1-picryl-hydrazyl-hydrate.

## Data Availability

The results are available from the authors.
